# Interactions between Microbial Food Safety and Environmental Sustainability in the Fresh Produce Supply Chain

**DOI:** 10.3390/foods10071655

**Published:** 2021-07-17

**Authors:** Francisco López-Gálvez, Perla A. Gómez, Francisco Artés, Francisco Artés-Hernández, Encarna Aguayo

**Affiliations:** 1 Postharvest and Refrigeration Group, Escuela Técnica Superior de Ingeniería Agronómica (ETSIA), Universidad Politécnica de Cartagena (UPCT), Paseo Alfonso XIII, 48, 30203 Cartagena, Spain; francisco.lopezgalvez@upct.es (F.L.-G.); fr.artes@upct.es (F.A.); fr.artes-hdez@upct.es (F.A.-H.); 2 Food Quality and Health Group, Institute of Plant Biotechnology (UPCT), Campus Muralla del Mar, 30202 Cartagena, Spain; perla.gomez@upct.es

**Keywords:** fruits, vegetables, life cycle assessment, food losses, pathogens, foodborne disease, One Health

## Abstract

Improving the environmental sustainability of the food supply chain will help to achieve the United Nations Sustainable Development Goals (SDGs). This environmental sustainability is related to different SDGs, but mainly to SDG 2 (Zero Hunger), SDG 12 (Responsible Production and Consumption), SDG 13 (Climate Action), and SDG 15 (Life on Land). The strategies and measures used to improve this aspect of the food supply chain must remain in balance with other sustainability aspects (economic and social). In this framework, the interactions and possible conflicts between food supply chain safety and sustainability need to be assessed. Although priority must be given to safety aspects, food safety policies should be calibrated in order to avoid unnecessary deleterious effects on the environment. In the present review, a number of potential tensions and/or disagreements between the microbial safety and environmental sustainability of the fresh produce supply chain are identified and discussed. The addressed issues are spread throughout the food supply chain, from primary production to the end-of-life of the products, and also include the handling and processing industry, retailers, and consumers. Interactions of fresh produce microbial safety with topics such as food waste, supply chain structure, climate change, and use of resources have been covered. Finally, approaches and strategies that will prove useful to solve or mitigate the potential contradictions between fresh produce safety and sustainability are described and discussed. Upon analyzing the interplay between microbial safety and the environmental sustainability of the fresh produce supply chain, it becomes clear that decisions that are taken to ensure fresh produce safety must consider the possible effects on environmental, economic, and social sustainability aspects. To manage these interactions, a global approach considering the interconnections between human activities, animals, and the environment will be required.

## 1. Introduction

Fresh produce is being increasingly recognized as a source of foodborne outbreaks [1,2]. The burden of such outbreaks includes economic losses, healthcare costs, loss of productivity, reductions in the quality of life, and mortality [3]. Using data from foodborne outbreaks that occurred between 1998 and 2018 in the USA, the Centers for Disease Control and Prevention linked a significant proportion of the illnesses caused by three key pathogens (*Salmonella*, *Escherichia coli* O157, and *Listeria monocytogenes*) to produce (including fruits, sprouts, vegetable row crops, and seeded vegetables) [4]. According to a report from World Health Organization (WHO) and Food and Agriculture Organization (FAO) [5], fruits and vegetables are among the main identified vehicles of foodborne Shiga-toxin-producing *E. coli* (STEC) illness. Regarding the economic losses, the study by Mackenzie and Apte attributed the costliest food recalls in the USA, involving losses of hundreds of millions of dollars, to fresh produce (tomato, spinach) [6]. As a consequence of such a situation, the fresh produce industry, governmental institutions, and researchers all around the world are seeking ways to guarantee the microbiological safety of such products. The Center for Produce Safety (Woodland, CA, USA) is an example of an organization managing this collaboration to fill knowledge gaps in the food safety of produce (https://www.centerforproducesafety.org/ accessed on 16 July 2021). In the present work, the term fresh produce includes fresh (not subjected to thermal treatment) unprocessed and minimally processed fruits and vegetables. Frozen or dried fruits and vegetables are not within the scope of this work, although some of the concepts and discussions presented are to a certain extent applicable to those food items.

The impact that all human activities (including efforts to ensure food safety) have on environmental, social, and economic sustainability needs to be assessed [7]. In the framework of the present review, the term “sustainability” refers mainly to environmental sustainability. However, whenever possible, information regarding other aspects of sustainability (economic, social) has been included. The topic of food safety is related to most of the United Nations Sustainable Development Goals (SDGs) [1], especially with SDG3, which deals with good health and well-being [8].

Unfortunately, the fresh produce supply chain stakeholders on certain occasions receive conflicting recommendations concerning environmental sustainability and guaranteeing food safety [9]. The hierarchy between safety and sustainability is clear, with priority for the former [10]. However, numerous studies have suggested that taking decisions considering only food safety leads to inefficient strategies that do not achieve the proposed goals and can have negative consequences in other aspects such as environmental sustainability [11,12,13]. A global approach including co-management for food safety and sustainability (not only environmental but also economic and social) is put forward as the best strategy to deal with the potential conflicts [8]. For example, in the primary production step, keeping areas with natural vegetation in the agricultural lands could help to maintain an equilibrium between food safety and environmental and economic concerns [12]. Decision-making tools that integrate the different aspects involved (food safety, food quality, energy and water consumption, and environmental and economic impacts) are needed for optimum management of the supply chain [11,14].

The present study aims to point out a number of potential conflicts between microbial food safety and environmental sustainability in the fresh produce supply chain. Furthermore, strategies and approaches with the potential to reconcile these two aspects—when needed—are presented. The main sources of information used for identifying such interactions and their potential solutions were the scientific literature and documents from international and governmental institutions.

## 2. Pre-Harvest Measures Taken for the Sake of Fresh Produce Microbial Safety: Efficacy and Consequences Regarding Sustainability

The potential conflicts between microbial food safety and environmental sustainability at the pre-harvest level will be illustrated using mainly examples of food safety measures undertaken in the USA in the last two decades and their consequences. Particularly since the *E. coli* O157:H7 outbreak linked to California-grown spinach from 2006, fresh produce growers in the USA have faced conflicting demands regarding food safety and environmental preservation, with priority being given to the former [15,16,17]. The measures taken for the sake of fresh produce microbial safety included the elimination of natural vegetation, reduction of the presence of wild animals in agricultural areas, avoidance of the use of manure-based amendments, and, more recently, the disinfection of irrigation water [18,19,20]. The following paragraphs provide details on the food safety measures applied and the conflicts with sustainability and detail current opinions regarding the outcomes and the way forward.

Farmers have been encouraged to remove natural vegetation to reduce the risks of wildlife intrusion [12]. However, it has not been confirmed whether the elimination of non-crop vegetation reduces the presence of pathogenic bacteria in the crops. For example, Karp et al. [16] did not find an increased prevalence of pathogenic (enterohemorrhagic *E. coli*, *Salmonella* spp.) and indicator bacteria (generic *E. coli*) on leafy green vegetables grown near areas with non-grazed non-crop vegetation. Sellers et al. [21], analyzing fecal samples from wildlife intruders (rodents), did not observe a higher risk of the presence of pathogenic microorganisms in agricultural fields (walnut, tomato) surrounded by hedgerows, compared to fields with controlled field edge vegetation. Smith et al. [22] detected a higher presence of *Campylobacter* spp. in avian fecal samples from crop farms (brassica plants) located in landscapes with high mammalian livestock densities, compared to farms located in landscapes with larger areas of natural habitat. Fonseca et al. [23], analyzing local birds that inhabited near leafy green growing fields in the U.S. southwest, reported the absence of *Salmonella* spp. and *E. coli* O157:H7. The presence of vegetation barriers surrounding agricultural fields can have benefits for the environment and also limit the wind dispersion of pathogenic bacteria [24]. Therefore, recent studies raise doubts as to whether limiting the presence of non-crop vegetation in farmlands does lead to safer fresh produce.

The use of organic amendments has positive effects on the health of agricultural soils (e.g., on soil microbiota functional diversity) [25,26]. On the other hand, the use of raw manure has been linked to a higher prevalence of pathogenic microorganisms in agricultural soils compared to the use of synthetic fertilizers [27]. Avoiding the use of biological soil amendments is one of the preventive strategies that has been proposed and used to reduce the food safety risk of fresh produce [20]. However, recent studies suggest that using properly treated animal-based manure should be reconsidered. Devarajan et al. [28], analyzing corn growing fields, concluded that the application of appropriately managed poultry litter could lead to a lower risk of the presence of *Salmonella* spp. and *Listeria monocytogenes* in farms. Those authors suggest that this organic manure would stimulate the presence of a thriving pathogen-inhibiting microbiota in the soil. Gu et al. [27] did not detect *Salmonella* spp. in samples from tomato fields fertilized using poultry litter ash. Further research should confirm whether the use of properly managed animal-based manure can enable the combination of waste reuse, the preservation of soil health, and an adequate level of food safety.

Current evidence does not demonstrate that organic farming provides produce that is less safe than that grown using conventional practices [29]. However, the higher environmental sustainability of organic farming is questioned. Although it can achieve a local reduction in the environmental impact, the economic and social aspects (e.g., food security) make organic farming an inadequate alternative to the predominant conventional farming systems [30]. To maintain current produce supply levels using mainly organic agriculture would demand a substantial increase in the area of land devoted to agricultural activities, with the risk of leading to an increase in greenhouse gas emissions [31].

Irrigation water is an important vector for the contamination of fresh produce (e.g., leafy crops) with pathogenic microorganisms [32,33]. Disinfection of irrigation water can be used as a preventive measure in those settings in which water presents a higher microbiological risk (e.g., when reclaimed urban wastewater is used for irrigation) [34,35,36]. However, risk–benefit assessments should consider potential negative effects regarding the presence of disinfection by-products (DBPs) in the crop. For example, the presence of chlorate (ClO^3−^) has been reported in leafy greens irrigated with water treated with chlorine [37], chlorine dioxide [38], and electrolyzed water [39]. In the mentioned studies, the levels of ClO^3−^ in the crop were above the current maximum residue levels (0.7 mg/kg for leaf vegetables) allowed in the European Union [40] only when the irrigation water was treated with chlorine dioxide. The accumulation of disinfection residues in the soil and the potential alterations in the soil microbiota should also be considered. Truchado et al. [41], for example, observed no relevant changes in the crop and soil microbiota in a baby spinach field irrigated with water treated using chlorine dioxide, suggesting that this treatment (as applied in their study) would be eco-compatible. Martínez-Sánchez and Aguayo [42] studied the effect of irrigation with ozonated water (0.35–0.40 mg/L of O_3_) on the quality of capsicum seedlings grown in the nursery and found a reduced microbial load in the water (*E. coli* and total *Enterobacteria*) as well as a decrease in the mesophilic load of capsicum seedlings.

**Figure 1:**
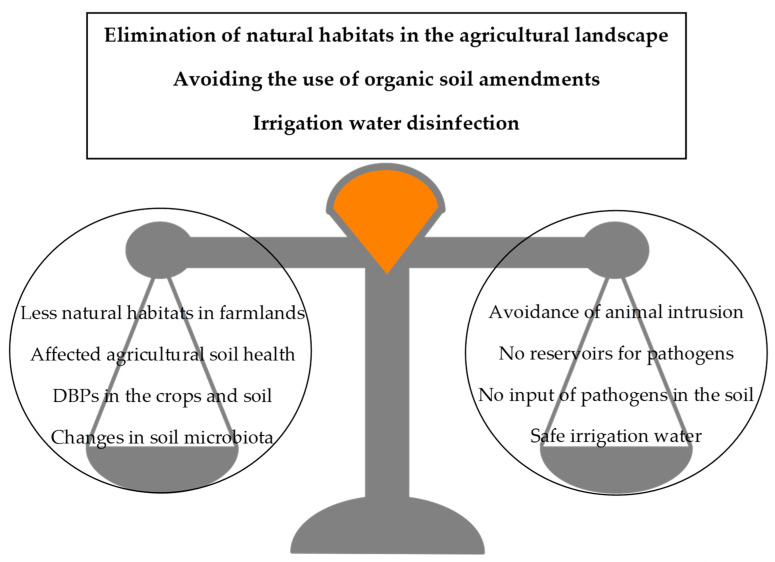
Potential environmental impacts (left) and fresh produce safety gains (right) of different measures applicable in primary production. DBPs: disinfection by-products.

Figure 1 summarizes the topics covered in this section. In conclusion, the effectiveness and the side-effects of the microbial food safety measures taken in the primary production step should be carefully assessed to determine opportunities for co-management of microbial safety and environmental sustainability.

## 3. Post-Harvest Management in the Fresh Produce Supply Chain and Interactions between Safety and Sustainability

### 3.1. Structure of the Fresh Produce Supply Chain

Some studies assign an important share of the environmental impact of fresh produce (e.g., tomato, apple) to the transport stage [43,44]. Current food transport systems cause the emission of greenhouse gases (e.g., methane, carbon dioxide, nitrous oxide), which are implicated in global warming. Transportation is also involved in other environmental impacts such as non-renewable energy use, terrestrial acidification, and freshwater eutrophication [43]. Consequently, shortening the food supply chains has been proposed as a strategy to increase the sustainability of the food industry [45]. However, while the positive effects of short food supply chains in social sustainability are clear, the impacts on economic and environmental sustainability are questionable [46]. Furthermore, concerns have also been raised about the safety standards of local supply chains [47]. According to Schmitt et al. [48], food safety is more closely monitored in products managed in large quantities in the global supply chains compared to that in local products. In the fresh fruit and vegetable supply chain, the size of the customer affects the safety management, with major retailers putting pressure on wholesalers regarding private certifications, thereby leading to greater safety [49]. In contrast, some consumers attribute higher food safety to short supply chains [50]. In developing countries, fresh produce companies oriented to the export market (therefore, involved in global supply chains) have more advanced management regarding food safety issues, as compared to smallholdings, which are oriented to the local market [51]. In many countries around the world, farmers’ markets or local food markets are popular settings that facilitate consumer access to local fresh produce. Despite concerns regarding the food safety procedures in such markets [52], the potential higher prevalence of microbial contamination in the products sold in farmers’ markets compared with other retailers remains controversial [47,53].

### 3.2. Water Reuse and Food Safety in the Fresh Produce Industry

The industrial handling, conditioning, and processing of fresh produce have an important water footprint, due to their considerable water demand and the generation of large quantities of wastewater [54,55]. Water can be used for cleaning, washing, disinfection and rinsing, transportation, blanching, cooling, or even heating the products [56,57,58,59,60]. Apart from water consumption, the use of water also involves the consumption of energy for cooling, heating, or pumping [61]. Mundi et al. [62] indicated that one kilogram of processed fruit and vegetables entails the generation of 5 L of wastewater; its characteristics depend on factors such as the type of processed product, and the configuration and management of the processing lines. In the case of fresh-cut produce processing plants, 2 to 11 m^3^ of good-quality water is consumed per ton of product [63], although a significant part of this water is commonly reconditioned and reused to reduce water consumption and wastewater generation [64]. However, environmentally beneficial water reuse can have consequences from the microbial food safety point of view, due to potential cross-contamination between batches [61]. Furthermore, it can also lead to the accumulation of disinfection by-products in the wash water [65]. Water treatment needs to be optimized to enable water reuse, whilst reducing the microbial and chemical safety risks [66,67]. In particular, reconditioning the water using physical treatments can be a sustainable alternative [55,68]. In any case, the presence of chemical antimicrobials is required to reduce the risk of cross-contamination by maintaining continuous disinfection processes in the washing tanks [69]. Apart from water reuse, the recovery of useful compounds from the fresh fruit and vegetable processing wastewater has been suggested as an approach to increase the sustainability of the industry [70]. However, the implementation of such a strategy faces different obstacles, including safety issues such as the potential presence of pathogenic microorganisms in the material recovered [71].

### 3.3. Packaging of Fresh Produce

Packaging is another aspect of the fresh produce supply chain in which conflicts between food safety and sustainability can appear. Guaranteeing food safety is one of the benefits of fresh produce packaging [72]. Nevertheless, packaging can also have drawbacks regarding microbial safety, such as the increased survival of pathogens in high-moisture environments (e.g., bagged lettuce) [73]. Regarding the packaging/sustainability interaction, the recommendations and regulations aimed at guaranteeing food safety tend to promote the utilization of single-use packaging [12], although multiple-use containers can be more sustainable. For example, the utilization of reusable plastic containers (RPCs) for the handling, transport, and commercialization of fruits and vegetables has the potential to improve the sustainability of the fresh produce supply chain [74]. However, although the use of these RPCs has never been linked directly with any fresh produce outbreak, a lack of hygiene can lead to unwanted risks [75,76,77]. On the other hand, at the consumer level, Barbosa et al. [78] detected diverse microorganisms, including pathogens, in multiple-use plastic bags utilized for food transportation. In the specific case of the controversial plastic packaging, it helps to reduce food waste in the fresh produce supply chain, thus improving sustainability [72]. However, the current life cycle of plastic packaging does not fit a circular economy approach. Consequently, policymakers should promote the utilization of alternative and sustainable packaging options, due to the importance of the packaging sector in the sustainability of the food supply chain [79,80].

### 3.4. Temperature Control

Control of storage temperatures during preservation is essential for maintaining the quality of fresh produce and, thereby, avoiding food waste and the associated impact on sustainability [56]. It also helps in guaranteeing the microbial safety of such products (e.g., leafy greens) [81]. However, storage temperature control entails a cost in energy consumption that affects the sustainability of the supply chain [11]. Tools for the optimization of temperature control in the fresh produce supply chain must consider safety, spoilage, and energy consumption [14]. New technological developments (e.g., Internet of Things, Artificial Intelligence, Big Data, Blockchains, etc.) are expected to improve the control of food cold-chain logistics in the coming years, with positive impacts on the safety and quality of fresh produce, reducing food waste and the environmental impact [82,83]. Wu et al. [84] suggested that a holistic approach combining life cycle assessment with virtual cold chains could help to design more sustainable fresh fruit cold chains. However, simpler changes could also have a significant impact. For example, the study by Xie et al. [85] suggests the use of closed displays for refrigerated fresh-cut leafy greens in retail shops as a solution that combines keeping the quality (avoiding food waste), microbial safety, and energy savings.

## 4. Relationship between Food Loss/Waste and Food Safety

Food loss is the decrease in the quantity or quality of food resulting from decisions and actions by food suppliers in the chain, excluding retailers, food service providers, and consumers. Food waste refers to the decrease in the quantity or quality of food resulting from decisions and actions by retailers, food service providers, and consumers [86]. The reduction of food loss/waste is included in the United Nations Sustainable Development Goals from the 2030 Agenda for Sustainable Development (SDG 12, Target 12.3) [1]. Food is lost/wasted in many ways: fresh produce that deviates from what is considered optimal, for example in terms of shape, size, and color, is often removed from the supply chain during sorting operations. Foods that are close to, at, or beyond the “best before” date are often discarded by retailers and consumers. Large quantities of wholesome edible foodstuffs are often unused or left over and discarded from household kitchens and eating establishments. Around one-third of the world’s food is lost or wasted every year [87]. This 2011 estimate by the FAO is in the process of being replaced by two separate indices: the Food Loss Index (FLI) and the Food Waste Index (FWI). The FLI provides new loss estimates from post-harvest up to, but not including, the retail stage.

The significant amount of the food produced that is lost or wasted entails an unnecessary environmental impact (greenhouse gas emissions, use of land and water resources) [88]. Food loss and waste includes not only the organic material but also the water and energy utilized for the production and the components of the packages [89]. A reduction in food loss/waste could improve the sustainability of fresh food, adding a sizable quantity to the global food supply, thereby reducing the need to intensify production in the future [90]. Less food loss and waste would lead to more efficient land use and better water resource management, with positive impacts on climate change and livelihoods (http://www.fao.org/food-loss-and-food-waste/flw-data accessed on 16 July 2021).

The recommendations to reduce food loss/waste should consider their viability (technical and economic), the constraints due to food quality and safety requisites, the point of view of society, and the environmental impacts [91]. Fresh produce supply chain stakeholders are receiving messages on the topics of food loss/waste and food safety that are, to a certain extent, contradictory [14,92]. On the one hand, measures that help to reduce food loss/waste could increase the food safety risk for the consumers [93]. On the other hand, the food safety policy should be well calibrated to avoid unnecessary food loss/waste [12]. For example, the confusion of consumers regarding the relationship between food safety and food date labels can lead to food waste [94]. In the European Union, perishable foods after the “use by” date shall be deemed to be unsafe and, consequently, their marketing is prohibited [95]. Conversely, the “best before” label informs about quality, not safety, but up to a quarter of the population thinks that food should not be eaten after that date [96].

Food waste management methods include (in order of priority) prevention (e.g., prediction of demand by consumers, planned food shopping), redistribution (for human consumption), valorization (e.g., industrial recycling of waste, its use to produce animal feed), and food waste treatment (e.g., composting, incineration) [93]. The redistribution of excess food for human consumption (e.g., donation) or its use to produce animal feed can help to reduce food waste [97]. Decentralization, lack of professionalization, insufficient or non-existent regulation, and lack of monitoring by authorities have been identified as important problems that make the optimization of food safety within food donation/acceptation chains difficult [98,99]. Hecht and Neff [100] indicated that future studies aimed at performing a risk–benefit assessment of food redistribution interventions need to include the effects on health, the environment, and the economy. Safety requirements are also essential when assessing the feasibility of the valorization of food waste [101]. To prevent microbial safety issues, food waste could require treatment (e.g., pasteurization) to enable its valorization as a food-grade ingredient [102]. The option of food waste treatment is appropriate for a wider range of food waste categories than redistribution and valorization [103], because fruits and vegetables recovered and reintroduced into the supply chain for human consumption constitute higher safety risks [104].

In the case of fresh produce, the use of different pre- and post-harvest tools can help to decrease food loss/waste. The effects of proper management, such as storage in well-ventilated rooms, storage in a controlled atmosphere, modified atmosphere, ethylene scavengers, proper temperature and relative humidity (RH), heat treatment, and others, plus different sustainable pre- and post-harvest treatments (i.e., natural compounds, ozone, ultraviolet irradiation, biocontrol agents), and their combinations are sustainable treatment methods that help to reduce the decay of fresh fruits and vegetables (e.g., carrot, spinach, peach, nectarine) [42,105,106,107,108,109,110].

## 5. Climate Change and Fresh Produce Safety

Modifications in the Earth’s atmospheric composition caused by human activities are driving climate change [111]. Increases in the mean air temperature and the frequency of extreme weather events are among the expected consequences of climate change [112]. These changes are associated with a potential risk of an increased presence of certain pathogenic microorganisms and toxins in food [113,114,115], including vegetables [116]. The work by Liu et al. [116] focusing on pre-harvest leafy green vegetables concluded that the rise in temperatures and modifications in precipitation patterns will affect the contamination sources and the pathways of pathogens, likely leading to an increase in the contamination of these products with pathogenic microorganisms. Foodborne pathogens are among the most climate-sensitive human pathogenic microorganisms [117]. Holvoet et al. [118] observed a positive correlation between the presence of pathogenic microorganisms in lettuce irrigation water and temperature. Extreme precipitation can cause flooding in agricultural fields, and the risks of using open-air areas after a flood event, where potential exposure to infective microbial contamination exists, must be evaluated [119]. Flooding has been associated with pathogenic contamination of leafy green vegetables [120]. Droughts are also expected to be more frequent and intense in the future in some areas of the planet that are currently affected by that phenomenon [121], and drought-stressed plants (e.g., lettuce) could be more susceptible to the internalization of pathogenic bacteria [122]. The potential impacts of climate change on the contamination of food with pathogenic microorganisms are complex, and knowledge gaps are numerous [123,124]. In any case, adaptation and mitigation strategies will need to be implemented to reduce the negative impacts of climate change on fresh produce safety [125,126]. The work by Kirezieva et al. [125] used experts’ opinions to evaluate potential responses to the impacts of climate change on fresh produce safety, concluding that strengthened control activities (e.g., water microbial quality monitoring, personal hygiene requirements) and improved guidance and training for farmers will be needed.

## 6. Approaches, Strategies and Solutions to Solve Conflicts between Fresh Produce Microbial Safety and Environmental Sustainability

Co-management at the farm level comprises balancing environmental protection with food safety and productivity goals [127]. Certain types of agricultural management, such as organic agriculture and, particularly, biodynamic agriculture, which fosters the diversity of plant and animal life, increase the health and resilience of the organism farm. Biodynamic farms aspire to generate their fertility through composting, integrating animals, cover cropping, and crop rotation [128].

The work by Crohn and Bianchi [129] identified the assessment of the fate of pathogenic microorganisms in farmlands as the most urgent research topic regarding the co-management of food safety and surface water quality. In the last two decades, the information available on the behavior of pathogenic microorganisms in agricultural settings has increased significantly, mainly based on controlled tests with the inoculation of lab-prepared pathogens [130]. The development of methods for the large-scale affordable detection of pathogenic microorganisms in the agricultural environment would be of enormous help in tracking the sources of produce-borne outbreaks, as well as in the assessment of the fate of pathogens [131]. Information obtained directly from the environment would provide us with a more reliable picture of the situation, which could then be used as a background for the development of recommendations and legislation. Currently, one of the tools assessed to detect fecal contamination in fresh produce in the growing fields is hyperspectral imaging. Cho et al. evaluated this technique for the on-site detection of fecal contamination in romaine lettuce, with positive results [132]. Until more detailed and complete information on this topic becomes available, a conservative approach is likely to be taken by the competent authorities and by supply-chain stakeholders to avoid outbreaks and the subsequent consequences on public health and the agrifood sector [133,134].

In many cases, the experimental studies focus on a topic with a narrow approach, without considering interactions with other aspects. For example, over the years, numerous studies have assessed the efficacy of antimicrobial treatments that are applicable to fresh produce, without considering aspects such as the economic and environmental sustainability of their usage. However, in the last decade, studies with a more global perspective have been performed. Vigil et al. [68] assessed sanitation and decontamination techniques for fresh-cut produce using a life cycle approach. Papoutsis and Edelenbos [106] reviewed different sustainable post-harvest treatments for carrots (considering both human health and the environment). On the topic of food-waste reduction, Tromp et al. [135] assessed the potential reuse of salads in salad bars considering safety and quality. Yam and Takhistov [136] also considered microbial safety, as well as economic and environmental sustainability, when assessing an alternative packaging technology for fresh produce. Looking to the future, both available and innovative technologies (e.g., nanotechnology) will help to make fresh produce safety and sustainability compatible [137].

Proper traceability is crucial in the management of the fresh produce supply chain to ensure safety and avoid loss and waste [34]. Being able to quickly trace back any contamination to its source can reduce food loss/waste by defining precisely which lots should be disposed of, thereby avoiding the unnecessary elimination of uncontaminated batches [6]. The suitability of the use of blockchain technology to enhance traceability (and therefore the safety and sustainability) of fresh produce is being assessed [83].

In the conflicts between sustainability and safety, from the political organization’s standpoint, there is a lack of a global perspective. Different departments, agencies, etc. have different and narrow-sighted ideas on how to deal with the issues of safety and sustainability (environmental, economic, and social) of the food supply chain [12,13]. Multiple criteria decision analysis has been suggested as a structured tool for decision-making in this complicated framework [14]. Regarding the concept of food safety, Leib and Pollans [12] proposed a more global view that should include not only the current concept (acute risks linked to ingestion of pathogens or toxins) but also other issues such as the health risks associated with cumulative ingestion and the health risks linked to the life cycle of food from production to end of life. Furthermore, measures aimed at improving fresh produce safety should be adapted taking the diversity of agrifood systems into account [7]. The One Health approach, based on the concept of the interconnection between human beings, animals, and the environment, promotes the formation of multidisciplinary teams that can work to obtain solutions to challenges that involve health, social, and environmental issues [8,138]. Finally, to promote the safety, security, and sustainability of the produce supply chain, the training and education of all the stakeholders are crucial [139]. Table 1 summarizes the topics covered, the potential implications on fresh produce microbial safety and environmental sustainability, and the optimization options covered in this review.

## 7. Conclusions

Clear conflicts arise when analyzing the interactions between environmental sustainability and microbial safety of the fresh produce supply chain. Although the safety aspect has priority, the decisions taken for the sake of fresh fruit and vegetable safety ought to consider the potential impacts on the whole sustainability (environmental, economic, and social). The present work provides examples of frictions between microbial safety and environmental sustainability in the fresh produce supply chain. The stated issues are present throughout the supply chain (pre- and post-harvest) and affect all the different stakeholders (from primary producers to consumers). A global approach to deal with these safety/sustainability interactions is required. Widening the concept of food safety, co-management, multicriteria decision analysis, technological advances (e.g., cold chain management), working in multidisciplinary teams, and training the stakeholders are some of the strategies and approaches that will help to deal with sustainability/safety conflicts. In this context, the concept of One Health applied to the fresh produce supply chain appears as a correct approach to analyze and make decisions aimed at solving these challenges.

## Figures and Tables

**Table 1:** Summary of topics covered, potential implications on fresh produce microbial safety and environmental sustainability discussed, and optimization options.

Topic	Sub-Topic	Microbial Safety	Environmental Sustainability	Optimization Options
Fresh produce safety at primary production	Elimination of natural habitat	Avoidance of animal intrusion	Affects wildlife and ecosystem services	Find co-management options
	Avoidance of animal-based organic amendments	Avoidance of input of pathogens	Loss of positive effects on soil health	Use of appropriately treated animal-based manure
	Irrigation water disinfection	Safer irrigation water	Input of chemicals in the agricultural environment (disinfectants and DBPs)	Use of environmentally friendly disinfection methods (e.g., ultraviolet irradiation).
Food waste	Food date labels	“Use by” date labels are needed for safety reasons	Misinterpreting “use by” and “best before” dates can increase food waste	Clarifying the meaning of food date labels; consumer education
	Food redistribution	Fruits and vegetables reintroduced into the food supply chain can increase the risk	Redistribution is an important food waste management method	Development of regulations; monitoring by authorities
Supply chain structure	Short supply chains	Concerns over safety standards of shorter chains	Potentially more sustainable due to reduced transport	Scientific statements on the safety in short versus global supply chains; development of regulations; monitoring by authorities
Climate change		Potential increments in the prevalence of some pathogens	Caused by unsustainable human activities	Research to fill knowledge gaps on the safety consequences of climate change
Water reuse		Requires water treatment to avoid microbial safety risks	Potential to increase the sustainability of the fresh produce industry	Optimization of process water management; identification of sustainable options
Temperature control		Needed for some products (e.g., fresh-cut)	Avoids food waste but demands energy	Strategies for energy saving to reduce environmental impact (e.g., supply chain optimization using IoT ^a^, AI ^b^, Big Data)
Packaging	Single-use packaging	Safer	Less sustainable	Renewable single-use packaging if needed

a: Internet of Things; b: Artificial Intelligence.
